# Kindergarten predictors of dyslexia pathways in Hebrew: a 5-year longitudinal study

**DOI:** 10.1007/s11881-025-00354-x

**Published:** 2025-11-25

**Authors:** Rotem Yinon, Dana Tal, Shelley Shaul, Yaniv Kanat-Maymon, Tami Katzir

**Affiliations:** 1 Edmond J. Safra Brain Research Center for the Study of Learning Disabilities, Department of Learning Disabilities, University of Haifa, 199 Aba Khoushy Ave., Mount Carmel, Haifa, 3498838 Israel; 2 Baruch Ivcher School of Psychology, Reichman University, Herzliya, Israel

**Keywords:** Dyslexia pathways, Hebrew, Morphological awareness, Early predictors, Late-emerging

## Abstract

While dyslexia is often considered a uniformly persistent condition, research has identified two additional developmental pathways: resolving and late-emerging dyslexia. Yet, most studies have focused on Western languages, and only a few have examined kindergarten predictors of these pathways, with morphological awareness largely overlooked. This five-year longitudinal study examined dyslexia pathways and their kindergarten predictors, including morphological awareness, in Hebrew—a Semitic language with distinct orthographic and morphological features. A cohort of 515 Hebrew-speaking children was followed from kindergarten through Grades 1 and 4. Dyslexia pathways were classified using dual cut-off criteria (≤ 16th percentile for dyslexia, ≥ 25th percentile for typical readers) based on word-reading fluency in Grades 1 and 4. Kindergarten measures included phonological awareness, letter knowledge, rapid automatized naming, and morphological awareness. Among children with dyslexia, three pathways were identified: persistent (35%), resolving (33%), and late-emerging (32%). Multinomial logistic regression, with typically developing readers as the reference group, revealed distinct kindergarten predictors. Letter knowledge deficits characterized all pathways. Phonological awareness predicted persistent and resolving dyslexia. Rapid naming predicted only persistent dyslexia, distinguishing it from resolving dyslexia. Morphological awareness uniquely predicted late-emerging dyslexia. These findings extend dyslexia pathway research to Semitic languages, showing how typological features shape pathway prevalence. They provide the first evidence that kindergarten morphological awareness deficits uniquely predict late-emerging dyslexia, demonstrating how the universal phonological-to-morphological shift in reading manifests in dyslexia trajectories and underscoring the need for comprehensive early screening approaches.

## Highlights

First comprehensive study of dyslexia pathways in Hebrew.

Three dyslexia pathways with comparable prevalence: persistent, resolving, and late-emerging.

Morphological awareness emerges as a key predictor of late-emerging dyslexia.

Demonstrates unique linguistic features’ impact on reading development and dyslexia.

## Introduction

Developmental dyslexia is characterized by persistent difficulties in accurate and/or fluent word reading despite adequate intelligence and educational opportunity (Lyon et al., 2003; Parrila & Protopapas, 2017), affecting approximately 7% of children worldwide, with prevalence estimates ranging from less than 5% to 20% depending on diagnostic criteria (Wagner et al., 2020; Yang et al., 2022). Longitudinal research has challenged the traditional view of dyslexia as a uniformly persistent condition, consistently documenting two additional developmental pathways: resolving and late-emerging dyslexia (Catts et al., 2012; Psyridou et al., 2020; Torppa et al., 2015). Despite these advances, most pathway research has been conducted in Western languages, leaving substantial gaps in our understanding of how language-specific features shape the manifestation of dyslexia across diverse linguistic contexts (Daniels & Share, 2018). Moreover, few studies have examined kindergarten predictors of these developmental pathways, which are critical for early identification and targeted intervention. Among these studies, morphological awareness has been largely overlooked as a potential early predictor, despite its well-established role in supporting efficient word recognition (Carlisle & Kearns, 2017; Castles et al., 2018). This omission is particularly significant in morphologically rich Semitic languages like Hebrew, where research highlights the central role of morphology from the earliest stages of reading acquisition (Bar-On & Ravid, 2011; Ravid & Schiff, 2006; Vaknin-Nusbaum et al., 2016) and demonstrates its effectiveness in intervention contexts (Vaknin-Nusbaum & Raveh, 2019; Vaknin-Nusbaum, 2021).

To address these gaps, this five-year longitudinal study examined dyslexia pathways and their kindergarten predictors within the distinctive linguistic context of Hebrew. As a Semitic language written in an abjad (consonantal) writing system (Shechter & Share, 2025), Hebrew provides an informative test case for understanding how typological features shape dyslexia trajectories. We tracked children’s word-reading fluency from Grade 1 to Grade 4 to identify distinct developmental pathways, in line with recent expert consensus that “across languages and age groups, difficulties in reading fluency and spelling are a key marker of dyslexia” (Carroll et al., 2025, p. 8). We then examined how four kindergarten language-related skills—phonological awareness, letter knowledge, rapid naming, and morphological awareness—predict membership in these pathways. The study aimed to improve early identification accuracy, inform targeted interventions, and advance a broader cross-linguistic framework for understanding the developmental nature of dyslexia.

### Long-term pathways of dyslexia

Although dyslexia has traditionally been viewed as a uniformly persistent condition (Parrila & Protopapas, 2017), longitudinal research has identified two additional developmental pathways. Late-emerging dyslexia (32–36% of cases) emerges after Grade 4 despite adequate early reading acquisition, whereas resolving dyslexia (6–28% of cases) is characterized by early reading difficulties that are no longer evident in later grades (Catts et al., 2012; Leach et al., 2003; Lipka et al., 2006; Psyridou et al., 2020; Torppa et al., 2015).

Despite growing recognition of these developmental pathways, research in this area faces both methodological and linguistic challenges. Methodologically, studies vary in classification criteria, using word-level accuracy (Catts et al., 2012; Lipka et al., 2006), fluency (Leach et al., 2003; Torppa et al., 2015), or both word-reading and reading comprehension measures (Catts et al., 2012; Leach et al., 2003; Psyridou et al., 2020). Cut-off thresholds also differ, ranging from stringent (10th percentile; Torppa et al., 2015) to more lenient (25th percentile; Lipka et al., 2006), with some studies adopting intermediate cut-offs (16th percentile; Leach et al., 2003; Catts et al., 2012) or using buffer zone methods to address borderline cases (Etmanskie et al., 2016; Psyridou et al., 2020). Linguistically, most research has been conducted in English, with relatively few studies examining more transparent orthographies such as Finnish (Psyridou et al., 2020; Torppa et al., 2015) or Dutch (de Bree et al., 2022). These script differences significantly influence both the manifestation and developmental course of dyslexia (Daniels & Share, 2018). In transparent orthographies with consistent grapheme-phoneme correspondences, decoding is typically acquired more easily (Caravolas et al., 2013; Seymour et al., 2003), and dyslexia more often manifests as slow, dysfluent reading rather than inaccuracy (de Jong & van der Leij, 2003; Landerl & Wimmer, 2000; Landerl et al., 1997). Accordingly, higher rates of resolving profiles have been reported in such languages compared to deep orthographies like English (Torppa et al., 2015).

Hebrew provides a particularly valuable context for investigating dyslexia pathways, as it is written in an abjad (consonantal) script with two orthographic versions that differ in transparency. Early reading instruction uses the pointed script, which includes vowel diacritics (nikud) that provide high phonological transparency through consistent, near-perfect symbol–sound correspondences (Share, 2017). By Grades 3–4, children transition to the unpointed script (without diacritics), the standard form of Hebrew, in which vowels are only partially represented by dual-function matres lectionis (AHWY letters), resulting in pervasive homography whereby almost every word can be read in multiple ways (Bar-On & Ravid, 2011; Share & Bar-On, 2018). These orthographic features may support resolving pathways through early facilitated decoding in the highly transparent pointed script (similar to patterns observed in Finnish; Torppa et al., 2015), while the transition to the opaque unpointed script in Grade 4 may trigger late-emerging reading difficulties in vulnerable children.

### Early language-related predictors of dyslexia pathways

Identifying early predictors of dyslexia pathways is crucial for timely and effective intervention (Partanen & Siegel, 2014; Verwimp et al., 2020), yet challenging due to the multifactorial nature of this disorder (Parrila & Protopapas, 2017; Pennington, 2006). Research consistently highlights key language-related skills—phonological awareness, letter knowledge, and rapid naming—that emerge prior to formal reading instruction and serve as critical building blocks for later reading achievement (Caravolas et al., 2013; Georgiou et al., 2012; Landerl et al., 2022). The relative importance of these skills varies across reading stages and languages (Castles et al., 2018; Landerl et al., 2022), suggesting that early risk factors may differentially predict distinct developmental pathways of dyslexia. Phonological awareness and letter knowledge are essential for basic decoding across orthographies (Caravolas et al., 2013). Phonological awareness shows stronger effects in deep orthographies and often diminishes after the early reading stages (de Jong & van der Leij, 1999; Georgiou et al., 2012; Ziegler et al., 2010), whereas letter knowledge tends to retain predictive power from kindergarten through both early and advanced stages (Leppänen et al., 2008; Yinon et al., 2025). Rapid naming has emerged as a robust universal predictor of reading fluency, with its contribution increasing over time (Araújo et al., 2015; Vaessen & Blomert, 2010).

Research indicates variation in how these early language-related skills predict distinct developmental pathways of dyslexia, although findings remain inconsistent across linguistic contexts. For example, in English, early phonological awareness deficits characterize both persistent and late-emerging dyslexia (Catts et al., 2012; Lipka et al., 2006), whereas in Finnish, persistent dyslexia is not marked by significant phonological impairments and late-emerging dyslexia is primarily linked to rapid naming deficits in kindergarten (Torppa et al., 2015). These cross-linguistic inconsistencies underscore the importance of investigating dyslexia pathway predictors within specific linguistic and orthographic frameworks.

### Morphological awareness as an early predictor of dyslexia pathways

While previous research has examined phonological awareness, letter knowledge, and rapid naming as predictors of dyslexia pathways (Catts et al., 2012; Lipka et al., 2006; Torppa et al., 2015), morphological awareness remains underexplored. This omission is significant given the universal developmental progression from phonological (or sub-morphemic) to lexical-morphological processing in reading acquisition (Share, 2025), which emphasizes the increasing importance of morphological skills in supporting efficient word recognition (Carlisle & Kearns, 2017; Castles et al., 2018) and their effectiveness in intervention contexts (Goodwin & Ahn, 2013), especially critical in deep orthographies, where grapheme-phoneme ambiguity increases reliance on morphological cues (Desrochers et al., 2018; Mousikou et al., 2020). This phonological-to-morphological shift suggests that pathway studies focusing solely on early phonological predictors risk overlooking key predictors of late-emerging dyslexia. Such oversight may reflect limited causal evidence for morphology as a primary deficit in dyslexia and its variable influence across languages and writing systems (Deacon et al., 2019; Landerl et al., 2022; Perfetti et al., 2019).

This gap is particularly pronounced in morphologically rich Semitic languages like Hebrew, which combine linear and non-linear root-and-pattern morphology within a complex, dense system of inflectional and derivational word formation. Unlike the linear affixation typical of Indo-European languages, most Hebrew content words are formed by interweaving tri-consonantal roots that carry core semantic meaning with vowel–consonant patterns that encode grammatical and prosodic information (Ravid & Schiff, 2006). This root-and-pattern structure is highly salient in print, often more so than in speech, and considered a cornerstone for understanding Hebrew orthography and acquiring reading skills (Frost, 2012; Ravid & Schiff, 2006; Share, 2017).

According to the Triplex Model (Share & Bar-On, 2018), Hebrew readers initially follow a sublexical path, relying primarily on phonological processing in the transparent pointed script during Grade 1, then gradually shift to lexico-morpho-orthographic processing in Grades 2–4, where morphology becomes essential for resolving phonological ambiguity during the transition to the unpointed script (Bar-On & Ravid, 2011; Frost, 2012). Empirical studies support this trajectory, showing that Hebrew-speaking children draw on multiple linguistic resources from the earliest stages, including both phonological and morphological knowledge (Bar-On & Ravid, 2011; Cohen-Mimran et al., 2023; Ravid & Schiff, 2006; Vaknin-Nusbaum et al., 2016), even during pointed-script reading that provides full phonological information (Cohen-Mimran et al., 2023; Yinon & Shaul, 2024), with a developmental shift toward greater morphological reliance between Grades 2 and 4 (Bar-On & Ravid, 2011; Yinon & Shaul, 2024). Furthermore, morphological deficits have been identified as an independent source of reading difficulty in Hebrew, beyond phonological impairments (Shechter & Share, 2025; Yinon & Shaul, 2025), and morphology-based interventions have proven effective for struggling readers (Vaknin-Nusbaum & Raveh, 2019; Vaknin-Nusbaum, 2021), reinforcing a causal role for morphology in this linguistic context (Shechter & Share, 2025).

Given this theoretical foundation, the phonological-to-morphological shift constitutes a universal hallmark of reading development, positioning morphological awareness as a potentially key predictor in dyslexia pathway research, though its timing and impact may vary according to language-specific features (Castles et al., 2018; Share, 2025). In Hebrew, early morphological skills may facilitate reading by leveraging the transparent root–pattern morphological system, potentially contributing to resolving pathways, while morphological deficits may underlie late-emerging difficulties due to increasing demands on morphological processing, particularly when navigating the unpointed script by Grade 4, as outlined in the Triplex Model (Share & Bar-On, 2018). By incorporating morphological awareness alongside established early predictors, this study addresses a critical gap, clarifying how the universal shift from phonology to morphology in reading interacts with language-specific features to shape distinct pathways of dyslexia.

### The present study

This longitudinal study investigates developmental pathways of dyslexia and their kindergarten predictors within the distinctive linguistic context of Hebrew. To identify dyslexia pathways, we tracked children’s word-reading fluency from Grades 1 to 4, since fluency difficulties are a key marker of dyslexia across ages and languages (Carioti et al., 2021; Carroll et al., 2025). Such difficulties may occur independently of, or result from, accuracy difficulties (Katzir et al., 2016) and are especially salient in transparent orthographies such as pointed Hebrew, where reading challenges manifest primarily as fluency rather than accuracy deficits (Share, 2017). The timeframe studied captures both early reading acquisition and the critical transition to the unpointed script (Share & Bar-On, 2018), aligning with the typical emergence of late-emerging dyslexia around Grade 4 (Catts et al., 2012; Torppa et al., 2015). Beyond the traditional early predictors examined in previous studies—phonological awareness, letter knowledge, and rapid naming—we also examined morphological awareness, which has not previously been investigated in dyslexia pathway research. This addition addresses a critical gap and offers a more comprehensive perspective on early risk factors, given the universal shift from phonological to morphological processing in reading development (Carlisle & Kearns, 2017; Castles et al., 2018; Share, 2025) and the particular importance of morphology in Semitic languages like Hebrew (Share, 2017; Shechter & Share, 2025).

Research questions:

1. What distinct developmental pathways of word-reading fluency emerge from Grades 1 to 4, and what are the prevalence rates of persistent, resolving, and late-emerging dyslexia in Hebrew?
2. To what extent do four kindergarten language-related skills predict children’s membership in these dyslexia pathways?

Consistent with previous research (Catts et al., 2012; Torppa et al., 2015), we expect to identify four distinct pathways of word-reading fluency from Grades 1 to 4: typically developing readers, persistent dyslexia, resolving dyslexia (where difficulties resolve by Grade 4), and late-emerging dyslexia (where difficulties emerge by Grade 4). We anticipate differential predictive patterns, with deficits in phonological awareness and letter knowledge predicting initial reading difficulties (resolving dyslexia), whereas deficits in rapid naming and morphological awareness are expected to predict late-emerging dyslexia, reflecting increasing reading demands and the orthographic and morphological complexity of Hebrew. The findings aim to advance theoretical understanding of both universal and language-specific aspects of dyslexia development and to inform evidence-based strategies for early identification and intervention across diverse linguistic contexts.

## Method

### Participants

A total of 515 Hebrew-speaking children (230 boys, 285 girls) participated in this study, followed longitudinally from kindergarten (mean age = 5.9 years, SD = 0.35) through Grade 1 (mean age = 6.8 years, SD = 0.50) to Grade 4 (mean age = 9.8 years, SD = 0.35). This cohort was part of a larger study of reading, writing, arithmetic, cognitive, and linguistic development in a representative Hebrew-speaking sample. The initial sample comprised 1,146 children recruited from 128 kindergartens in and around the greater Haifa region of northern Israel, covering a wide socioeconomic range across diverse neighborhoods and including both regular (secular) and religious educational settings. Ultra-Orthodox institutions were excluded due to substantial differences in curriculum, instructional practices, and cultural context, and special education settings were excluded to avoid confounding influences related to developmental disorders. While these exclusions may limit generalizability, they were necessary to accurately characterize dyslexia trajectories in the broader Hebrew-speaking population. Within included settings, no children were excluded based on developmental, learning, or attentional disorders. All participants demonstrated age-appropriate nonverbal abilities as assessed by Raven’s Progressive Matrices (Raven et al., 1998).

Substantial attrition occurred over the 5-year study period. Data collection was discontinued in mid-March of Grade 1 due to COVID-19-related school closures and resumed only at the end of Grade 3, resulting in a loss of 206 participants (18.0%). Additional attrition was due to school transfers or relocation (242; 21.1%), parental or child refusal (108; 9.4%), incomplete testing (51; 4.5%), transfer to special education (20; 1.7%), and other reasons (4; 0.3%). The final analytic sample comprised 515 children with complete data across kindergarten, Grade 1, and Grade 4. Attrition analyses revealed no significant differences between retained and excluded participants for kindergarten measures, including initial consonant deletion (*t* = 0.63, *p* =.526, *d* = 0.04), rapid naming–objects (*t* = 0.81, *p* =.416, *d* = 0.05), rapid naming–colors (*t* = 1.09, *p* =.278, *d* = 0.07), verb derivation (*t* = 0.85, *p* =.397, *d* = 0.05), adjective derivation (*t* = 0.89, *p* =.373, *d* = 0.06), and noun pluralization (*t* = 0.63, *p* =.526, *d* = 0.04). Minor differences were observed for final consonant deletion (*t* = 2.07, *p* =.039, *d* = 0.13; retained M = 4.35, SD = 4.02; excluded M = 3.84, SD = 4.04), letter identification (*t* = 2.95, *p* =.003, *d* = 0.18; retained M = 7.88, SD = 2.39; excluded M = 7.42, SD = 2.73), and letter naming (*t* = 2.41, *p* =.016, *d* = 0.15; retained M = 6.39, SD = 2.79; excluded M = 5.96, SD = 3.07). All effect sizes were below Cohen’s threshold for a small effect (d < 0.2), indicating that attrition was largely random and unlikely to bias the final sample. Missing data within measures were handled using maximum likelihood estimation.

### Measures

Composite scores for all constructs at each time point were computed by averaging the raw scores of the individual tasks. The internal consistency of these composites was evaluated using both intercorrelations among component tasks and Cronbach’s alpha.

### Word-reading fluency measures

Grade 1. Word-reading fluency was assessed using two timed reading tasks adapted from the Dutch One Minute Test (Brus & Voeten, 1973). In the first task, participants read aloud a list of 60 isolated pointed real words containing all consonants but only the/ɑ/vowel sign, which is typically the first vowel children acquire and master during this developmental period (α = 0.97). In the second task, participants read aloud 30 isolated pseudowords of varying lengths, all containing only the vowel/ɑ/and constructed to preserve Hebrew’s morphological structure (α = 0.94). Both tasks had a 60-second time limit with errors recorded. Scores reflected the number of correctly read words within the time limit. A composite score was computed by averaging the raw scores of the two tasks, which demonstrated a strong intercorrelation (*r* =.79, *p* <.001).

Grade 4. Word-reading fluency was assessed using two timed reading tasks from the Hebrew adaptation (Schiff et al., 2006) of the Test of Word Reading Efficiency (TOWRE; Wagner et al., 2001). Participants read aloud two separate lists—one pointed and one unpointed—each containing 104 different real words arranged in four columns and organized by increasing difficulty, number of syllables, phonological structure, length, frequency, and morphological complexity (α = 0.94). Both tasks had a 45-second time limit. Scores reflected the number of correctly read words within the time limit. A composite score was computed by averaging the raw scores of the two tasks, which demonstrated a strong intercorrelation (*r* =.82, *p* <.001).

### Kindergarten Language-Related measures

All measures described below were developed for this longitudinal study, with data collection beginning in kindergarten in May-June 2019 (see Procedure section for complete timeline).

*Phonological awareness* was measured using two tasks (Share et al., 2018; see Cohen-Mimran et al., 2023 for complete development details). In the initial consonant isolation task (CCVC words), participants were asked to repeat a spoken target word and then to isolate the initial consonant (e.g., say/dvaʃ/‘honey’ without/d/) (α = 0.84). In the final consonant isolation task (CVC words), they were asked to isolate the final consonant (e.g., say/bat/‘girl’ without/t/) (α = 0.80). For both tasks, after one demonstration and four training items, participants were presented with 10 target items, with one point awarded for each correct answer. A composite phonological awareness score was computed by averaging the raw scores from these tasks, which demonstrated a moderate-to-strong intercorrelation (*r* =.60, *p* <.001). Children scoring at or below the 16th percentile on this measure were classified as at-risk.

*Letter knowledge* was measured using two tasks (Share et al., 2018). In the letter identification task, participants were asked to identify a target letter from four presented letters across 10 items. The total number of correctly identified letters was computed (α = 0.82). In the letter naming task, participants were tasked to name 10 Hebrew letters presented individually on A4 paper. The total number of correctly named letters was computed (α = 0.87). A composite letter knowledge score was computed by averaging the raw scores from these tasks, which demonstrated a strong intercorrelation (*r* =.80, *p* <.001). Children scoring at or below the 16th percentile on this measure were classified as at-risk.

*Rapid automatized naming* was measured using two tasks adapted to Hebrew from Denckla and Rudel (1974). Participants were asked to name as rapidly as possible a matrix of 50 common objects (cat, table, chair, bee, and flower), and a matrix of 50 colored circles (red, yellow, blue, green, and black). For both tasks, the items were arranged randomly in 10 rows of five and children’s familiarity with the items was confirmed before testing. Total naming time (in seconds) was used as the score (test re-test reliability: 0.77 based on our sample), with lower scores indicating better performance. A composite rapid naming score was computed by averaging the raw scores from these tasks, which demonstrated a moderate intercorrelation (*r* =.45, *p* <.001). Children scoring at or below the 16th percentile on this measure were classified as at-risk.

*Morphological awareness* was measured using three tasks (Cohen-Mimran et al., 2018), with evidence of their validity documented in subsequent publications analyzing this same dataset (e.g., Cohen-Mimran et al., 2023; Cohen-Mimran & Share, 2025). These tasks were systematically adapted to create age-appropriate paradigms suitable for kindergarteners, with piloting procedures ensuring developmental appropriateness (see Cohen-Mimran et al., 2023, for complete details on development). In the resultative adjective derivation task, participants were required to derive 10 adjectives from spoken verbs, with two practice items provided (e.g.,/XaTXu/(They cut) the apple. Now the apple is __________/XaTuX/, ‘cut’) (α = 0.74). In the verb derivation task, participants were asked to derive eight verbal patterns from noun roots, with one modeling and one practice item (e.g., “What do we do with the/meXaDeD/(pencil sharpener)? With the pencil sharpener/meXaDeDim/“) (α = 0.75). In the noun pluralization task, participants were asked to produce 15 plural forms from singular nouns (e.g., “Here is a ball/kadur/(regular masculine noun). These are many_____/kadurim/(balls), including five regular forms (three masculine, two feminine) and eight irregular forms (six masculine, two feminine). Irregular suffixes take plural markers of the opposite gender, and items included both unaltered stems and stem-internal changes. Two practice items were provided (α = 0.77). For all three tasks, correct answers received positive reinforcement and repetition, while incorrect or no answers were corrected by the tester. Each correct response was awarded one point. A composite morphological awareness score was computed by averaging the raw scores from these tasks, which demonstrated a moderate-to-strong intercorrelation ranging from r = 0.58 to 0.65 (*p* <.001). This composite approach is supported by Principal Components Analysis showing these tasks load on a single factor accounting for 73.8% of variance (Cohen-Mimran et al., 2023), consistent with a unified morphological awareness construct. Children scoring at or below the 16th percentile on this measure were classified as at-risk.

### Non-verbal general ability (Grade 1)

The colored version of Raven’s Standard Progressive Matrices was utilized to evaluate non-verbal ability (Raven et al., 1998). Following an explicit demonstration, participants were asked to choose the missing part of a geometric pattern from various alternatives. All three sets were administered in this test (18 items in total).

### Procedure

Ethical approval for this study was granted by the Ministry of Education – Office of the Chief Scientist (permit #9667) and the Institutional Review Board at the Faculty of Education at the University of Haifa. The recruitment process involved obtaining cooperation from kindergarten/school principals and securing parental consent. Data collection occurred at three time points: language-related measures were administered at the end of kindergarten (May-June 2019), word reading abilities were assessed in mid-first grade (January-March 2020, before the COVID-19 pandemic outbreak), and again in mid-to-late fourth grade (March-June 2023).

All measures were administered individually across two sessions on different days within the same week, as part of a broader battery that included language, arithmetic, executive functions, reading, and writing tasks. Each session lasted approximately 30–40 min, depending on the child’s pace, and took place in a quiet room at the kindergarten or school. Given the longitudinal design, different research assistants administered the measures at each time point. All received identical, standardized training at the Faculty of Education, including protocol instruction, supervised practice, and ongoing oversight to minimize potential examiner-related variability. Examiners held backgrounds in education, psychology, or speech-language pathology. Scripted administration protocols were used for all tasks to ensure procedural fidelity across time points and examiners.

### Analytical strategy

To identify developmental pathways of dyslexia, we employed a classification approach using dual cut-off points (creating a buffer zone) based on recent methodological insights (Psyridou et al., 2020). Consistent with prior research, we defined the 16th percentile as the lower threshold for dyslexia, approximating one standard deviation below the mean and aligning with intermediate cut-offs commonly used in the field (Leach et al., 2003; Catts et al., 2012). The 25th percentile served as the upper threshold for typical reading performance (Psyridou et al., 2020).

This buffer zone approach addresses a key limitation of traditional single cut-off methods, which often oversimplify reading difficulties and risk misclassifying children with borderline performance. For example, under a single cut-off model, a child scoring at the 15th percentile in Grade 1 and the 17th percentile in Grade 4 might be categorized as “resolving,” despite showing minimal actual progress. In contrast, our method requires a more substantive gain—surpassing the 25th percentile—to classify a child as truly resolving. Psyridou et al. (2020) demonstrated through simulation-based analyses that buffer zones effectively control both measurement error effects and the arbitrariness of single thresholds, particularly critical when investigating unstable reading trajectories, like late-emerging and resolving dyslexia, where minor score fluctuations can yield misleading interpretations of developmental change. Using this classification scheme, we identified four distinct developmental profiles based on word-reading fluency performance in Grades 1 and 4:

- Typically developing readers (≥ 25th percentile in both grades).
- Resolving dyslexia (≤ 16th percentile in Grade 1 and ≥ 25th percentile in Grade 4).
- Late-emerging dyslexia (≥ 25th percentile in Grade 1 and ≤ 16th percentile in Grade 4).
- Persistent dyslexia (≤ 16th percentile in both grades).

To examine the mechanisms underlying these developmental pathways, we first determined the proportion of children in each dyslexia profile who exhibited risk-level performance on each kindergarten measure and conducted pairwise comparisons between each dyslexia group and the typically developing group to assess statistical significance. We then conducted multinomial logistic regression analyses using the typically developing readers as the reference group and calculated odds ratios to estimate the likelihood of profile membership based on kindergarten skill deficits.

## Results

### Descriptive statistics and correlations

Descriptive statistics are presented in Table 1, and correlations among the research variables are shown in Table 2. All kindergarten language-related measures were significantly associated with word-reading fluency in both Grade 1 and Grade 4. Phonological awareness showed a strong correlation with early reading in Grade 1 (*r* =.47, *p* <.001), which decreased by Grade 4 (*r* =.22, *p* <.001). Letter knowledge demonstrated stable, strong correlations across both time points (Grade 1: *r* =.43; Grade 4: *r* =.41; both *p* <.001). Rapid naming was negatively correlated with word reading at both grades (Grade 1: *r* = −.33; Grade 4: *r* = −.34; *p* <.001), indicating that slower naming speed consistently hindered reading performance. Morphological awareness displayed stable, modest correlations across both grades (*r* =.29, *p* <.001).

**Table 1:** Descriptive statistics: Means (M), Standard Deviations (SD), Min, Max, Skewness, Kurtosis (*N* = 515)

Variables	M	SD	Min	Max	Skewness	Kurtosis
Phonological Awareness	4.61	3.48	0	10	0.11	−1.41
Rapid Naming	74.32	19.98	35	194	1.43	3.28
Letter Knowledge	7.13	2.41	1	10	− 0.61	− 0.91
Morphological Awareness	6.63	2.22	0	10	− 0.53	− 0.45
Word-Reading Fluency Grade 1	17.08	8.7	0	44	0.24	− 0.15
Word-Reading Fluency Grade 4	44.61	12.72	6	83	0.04	− 0.18

Note. All variables represent composite scores created by averaging raw individual task scores

**Table 2:** Pearson correlation coefficients between measures (*N* = 515)

Variable	1	2	3	4	5
1. Phonological Awareness	-				
2. Rapid Naming	− 0.20	-			
3. Letter Knowledge	0.40	− 0.27	-		
4. Morphological Awareness	0.35	− 0.29	0.34	-	
5. Word-Reading Fluency Grade 1	0.47	− 0.33	0.43	0.29	-
6. Word-Reading Fluency Grade 4	0.22	− 0.34	0.41	0.29	0.56

Note. All correlations significant at *p* <.001 (2-tailed). For rapid naming, negative correlations indicate faster naming speed associated with better performance

### Classification of developmental dyslexia pathways

Applying the dual cut-off classification approach (16th and 25th percentiles), 442 children (85.8% of the total sample of 515) were classified into distinct reading profiles. Children with scores between the 16th and 25th percentiles (*n* = 73, 14.2%) were excluded to ensure clear classification boundaries. Four reading development profiles emerged (Table 3): typically developing readers (76%, *n* = 335), persistent dyslexia (8%, *n* = 38), resolving dyslexia (8%, *n* = 35), and late-emerging dyslexia (8%, *n* = 34). Among the children with dyslexia (24% of the analytic sample), the distribution across developmental pathway was relatively balanced: persistent (35.5%), resolving (32.7%), and late-emerging (31.8%).

**Table 3:** Classification into dyslexia pathways based on word-reading fluency at Grades 1 and 4

Word-Reading fluency		Grade 4		
		Above 25th percentile	Below 16th percentile	Total
Grade 1	Above 25th percentile	335	34	369
	Below 16th percentile	35	38	73
	Total	370	72	442

Note. Classification based on dual cut-off points (a buffer zone) at the 16th and 25th percentiles

### Kindergarten language-related predictors of dyslexia pathways

**Fig. 1:**
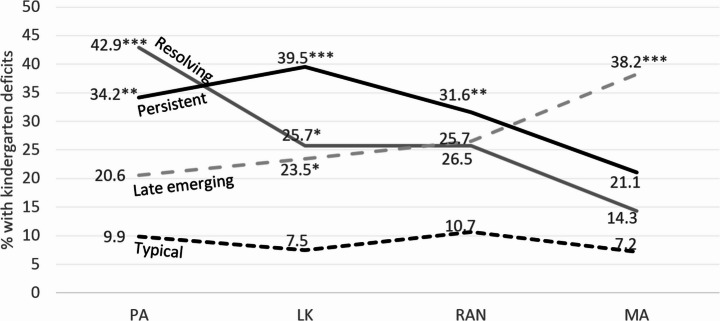
Percentage of children with kindergarten skill deficits within each dyslexia pathway. *Note*. PA = phonological awareness; LK = letter knowledge; RAN = rapid automatized naming; MA = morphological awareness. Asterisks indicate significant group differences compared to typical readers (*p <.05, **p <.01, ***p <.001)

Using typically developing readers as the reference group, risk-level performance on kindergarten language-related skills varied systematically across dyslexia pathways, with pathway-specific significant differences observed (Fig. 1). Multinomial logistic regression was then used to predict pathway membership from these measures. All four predictors were statistically significant overall: phonological awareness (*χ²* = 18.65, *p* <.001), letter knowledge (*χ²* = 16.64, *p* <.001), rapid naming (*χ²* = 8.94, *p* =.034), and morphological awareness (*χ²* = 16.64, *p* <.001). Model fit was acceptable (McFadden R² = 0.12; Cox & Snell R² = 0.18; Nagelkerke R² = 0.22).

Distinctive predictive patterns emerged for each dyslexia pathway (Table 4; Fig. 1). Letter knowledge deficits predicted all three pathways. Phonological awareness deficits significantly predicted both resolving and persistent dyslexia, whereas rapid naming deficits specifically predicted the persistent pathway. Morphological awareness deficits uniquely predicted late-emerging dyslexia. Interpretively, children with phonological deficits had over five times the odds of following a resolving dyslexia pathway (OR = 5.28, 95% CI [2.39, 11.65]). Letter knowledge deficits were associated with more than sixfold increased odds of persistent dyslexia (OR = 6.47, 95% CI [2.89, 14.50]). Morphological awareness deficits similarly conferred over sixfold increased odds of late-emerging dyslexia (OR = 6.21, 95% CI [2.68, 14.42]). Rapid naming deficits were linked to a threefold increase in odds for persistent dyslexia (OR = 3.10, 95% CI [1.36, 7.08]). These effect sizes indicate strong and pathway-specific predictive relationships.

**Table 4:** Prediction of dyslexia pathways based on kindergarten language-related measures

Kindergarten Measures	OR	b	SE	Wald	*p*	95%CI
	Late-emerging Dyslexia (*n* = 34)					
Phonological Awareness	1.47	0.39	0.50	0.60	0.438	[0.56, 3.89]
Letter Knowledge	2.89	1.06	0.49	4.78	0.029	[1.12, 7.47]
Rapid Naming	2.16	0.77	0.46	2.85	0.092	[0.88, 5.31]
Morphological Awareness	6.21	1.83	0.43	18.07	< 0.001	[2.68, 14.42]
	Resolving Dyslexia (*n* = 35)					
Phonological Awareness	5.28	1.66	0.40	16.97	< 0.001	[2.39, 11.65]
Letter Knowledge	3.19	1.16	0.47	6.24	0.012	[1.28, 7.94]
Rapid Naming	2.20	0.79	0.45	3.07	0.080	[0.91, 5.34]
Morphological Awareness	1.32	0.28	0.56	0.25	0.619	[0.44, 3.99]
	Persistent Dyslexia (*n* = 38)					
Phonological Awareness	3.00	1.10	0.42	6.87	0.009	[1.32, 6.82]
Letter Knowledge	6.47	1.87	0.41	20.59	< 0.001	[2.89, 14.50]
Rapid Naming	3.10	1.13	0.42	7.22	0.007	[1.36, 7.08]
Morphological Awareness	2.04	0.72	0.49	2.09	0.148	[0.78, 5.39]

Note. Reference group = typically developing readers. 95% CIs for significant predictors all exceed 1.0

## Discussion

This five-year longitudinal study investigated developmental pathways of dyslexia and their kindergarten language-related predictors within the distinctive orthographic and morphological context of Hebrew. We addressed two key research questions: (1) What distinct developmental pathways of word-reading fluency emerge from Grades 1 to 4, and what are the prevalence rates of persistent, resolving, and late-emerging dyslexia in Hebrew? (2) To what extent do kindergarten language-related skills predict membership in these pathways? Extending pathway research to a Semitic language, our findings reveal how typological features influence pathway prevalence and highlight the role of morphological awareness as a novel predictor of late-emerging dyslexia.

### Dyslexia pathways in Hebrew

Addressing our first research question, we identified four distinct word-reading fluency trajectories from Grades 1 to 4: typically developing readers and three dyslexia pathways—persistent, resolving, and late-emerging. Among children with dyslexia, the prevalence of the late-emerging pathway (31.8%) aligned with cross-linguistic findings (32–36%; Catts et al., 2012; Leach et al., 2003; Lipka et al., 2006; Torppa et al., 2015). The rate of persistent dyslexia (35.5%) was slightly lower than previously reported (approximately 40%; Catts et al., 2012; Torppa et al., 2015), whereas the resolving rate (32.7%) was higher than typically observed in the literature, more closely resembling rates reported in transparent orthographies like Finnish (27–28%; Psyridou et al., 2020; Torppa et al., 2015) than in English (6–12%; Catts et al., 2012; Leach et al., 2003).

These pathway prevalence differences can be attributed to both methodological and linguistic factors (Psyridou et al., 2020; Torppa et al., 2015). Methodologically, identification patterns are likely influenced by variations in cut-off criteria, word-reading measures (accuracy versus fluency), and follow-up periods ranging from Grade 4 (Lipka et al., 2006) to Grades 8 (Torppa et al., 2015) or 10 (Catts et al., 2012), reflecting developmental shifts in reading demands. Linguistically, two features of Hebrew may contribute to the observed patterns. First, Hebrew’s transparent pointed script in the early grades provides consistent letter-sound correspondences that facilitate decoding skill acquisition, enabling more children to overcome initial reading difficulties (de Jong & van der Leij, 2003; Landerl & Wimmer, 2000). Similar to Finnish, another highly transparent orthography, this transparency supports higher rates of resolving dyslexia than typically observed in English (Psyridou et al., 2020; Torppa et al., 2015). A second contributing factor may be Hebrew’s Semitic morphological system, characterized by transparent root-pattern structures explicitly represented in the writing system and critical from the earliest stages of reading acquisition (Bar-On & Ravid, 2011; Cohen-Mimran et al., 2023; Yinon & Shaul, 2024). This morphological transparency provides a valuable support framework, enabling children to leverage salient morphological regularities in print to overcome reading challenges. Together, the interplay of orthographic transparency and the root–pattern morphological structure likely increases resolving cases and reduces persistent ones, as Hebrew-speaking children benefit from multiple supportive mechanisms that contribute to the differing rates observed in our sample.

While Western languages like English or Finnish are consistently either deep or transparent, Hebrew uniquely requires children to shift between two orthographic versions that differ in transparency: from fully vowelized (pointed) to unvowelized (unpointed) script. Despite this added challenge in Grade 4, the prevalence of late-emerging dyslexia in Hebrew aligns with cross-linguistic rates, suggesting that this pathway reflects universal developmental difficulties. These findings imply that most Hebrew readers successfully navigate the transition, and its impact may be more limited than previously assumed. One reason may be that the unpointed script is considered opaque due to the missing vowel representation, rather than the inconsistent letter–sound correspondences typical of English (Cohen-Mimran & Share, 2025). Furthermore, children learning to read in morphologically rich Semitic systems may prioritize morphological transparency (root–pattern structures) over phonological transparency (vowel diacritics) (Yinon & Shaul, 2024).

### Kindergarten predictors of dyslexia pathways in Hebrew

Our second research question examined how kindergarten language-related skills predict dyslexia pathways within Hebrew’s linguistic context. The analysis revealed distinct predictive patterns for each pathway, indicating early markers identifiable before formal reading instruction. Children with persistent dyslexia exhibited multiple deficits across phonological awareness, letter knowledge, and rapid naming. Those with resolving dyslexia showed specific deficits in phonological awareness and letter knowledge. Notably, children with late-emerging dyslexia were uniquely characterized by deficits in morphological awareness and letter knowledge—a novel finding that meaningfully extends our understanding of this pathway. Thus, while letter knowledge deficits were common across all pathways, the distinct combinations of other deficits suggest unique underlying mechanisms, supporting a multi-componential view of dyslexia (Parrila & Protopapas, 2017; Pennington, 2006).

The predictive power of kindergarten letter knowledge deficits across all dyslexia pathways underscores its enduring and foundational role through both initial and advanced reading stages (Leppänen et al., 2008; Yinon et al., 2025), though it alone cannot differentiate between pathways. The phonological awareness deficit characteristic of the resolving pathway aligns with findings from transparent orthographies, where its impact is primarily on initial reading but diminishes over time (de Jong & van der Leij, 2003; Landerl & Wimmer, 2000). In Hebrew’s pointed script, with near-perfect symbol–sound correspondence (Share, 2017), children in the resolving group typically overcome early phonological deficits after Grade 1 (see also Yinon & Shaul, 2025). Rapid naming emerged as a key differentiator between the resolving and persistent groups, despite their shared deficits in phonological awareness and letter knowledge, reinforcing its increasing importance in predicting long-term reading fluency development (Araújo et al., 2015; Vaessen & Blomert, 2010). The shared phonological deficits observed in both resolving and persistent pathways suggest that phonological weaknesses constitute a core foundational deficit underlying initial reading difficulties, regardless of later developmental trajectory. However, the divergent outcomes highlight the multifactorial nature of dyslexia and suggest that phonological deficits alone are insufficient to determine long-term reading success; additional factors, such as rapid naming abilities (which contribute to the ‘double deficit’ pattern; Wolf & Bowers, 1999), distinguish children who overcome early difficulties from those who continue to struggle, at least in the Hebrew context.

In contrast to the phonological deficits observed in the persistent and resolving pathways, children in the late-emerging group exhibited a distinct pattern of early morphological deficits with a large effect size (OR = 6.21), suggesting that these children, despite having intact phonological awareness and thus successfully acquiring basic reading skills initially, encounter difficulties as reading demands shift toward morphological processing. This pattern aligns with theoretical frameworks positing a developmental progression from phonological to morphological processing (Carlisle & Kearns, 2017; Castles et al., 2018), as reflected in both the Hebrew-specific Triplex Model (Share & Bar-On, 2018) and Share’s (2025) universal Combinatorial Model. Our results demonstrate how this phonological-to-morphological shift manifests within dyslexia trajectories, providing the first prospective evidence linking early morphological deficits to late-emerging dyslexia. This addresses a key challenge in pathways research, given the difficulty of identifying children at risk for the late-emerging pathway (Catts et al., 2012; Psyridou et al., 2020; Torppa et al., 2015).

Importantly, while our findings indicate that kindergarten morphological awareness deficits predict late-emerging dyslexia by Grade 4—when the transition to unpointed script is complete—this should not be interpreted as suggesting that morphology only becomes important at this later stage. Research in Hebrew consistently shows that morphological knowledge supports reading from the earliest stages, even during pointed-script reading that provides full phonological information (Cohen-Mimran et al., 2023; Vaknin-Nusbaum et al., 2016; Yinon & Shaul, 2024). This early sensitivity reflects Hebrew’s transparent morpho-orthographic structure and the deep morphological foundations of Hebrew-speaking children, who encounter salient root-and-pattern structures from the earliest stages of language acquisition (Frost, 2012; Ravid & Schiff, 2006). Consistent with the Triplex Model (Share & Bar-On, 2018), these features accelerate the universal phonological-to-morphological progression, producing an earlier and more pronounced shift toward lexico-morpho-orthographic processing during Grades 2–4 (Yinon & Shaul, 2024). Thus, while the developmental principle itself is universal, its timing and magnitude are language-specific (Frost, 2012; Perfetti & Verhoeven, 2017). Accordingly, our study’s unique contribution is showing that morphological awareness deficits in kindergarten prospectively predict late-emerging reading difficulties years before they surface. However, given robust evidence for the early and foundational role of morphology in Hebrew, such predictive patterns may emerge well before Grade 4. Together, these findings underscore the importance of incorporating morphological awareness into early screening frameworks across languages, while recognizing that its manifestations and predictive strength likely vary across linguistic contexts and warrant further investigation.

### Implications and Limitations

This study offers valuable theoretical and practical implications. At the theoretical level, it extends prior evidence on dyslexia pathways from English, Finnish, and Dutch to encompass Semitic languages, advancing understanding of both universal and language-specific aspects of dyslexia and reading development, with particular relevance for other morphologically rich languages, such as Arabic, that share similar linguistic features. First, our findings support a multi-componential view of dyslexia (Pennington, 2006), demonstrating that diverse early cognitive-linguistic deficits may give rise to distinct developmental pathways. Second, the contrast between resolving phonological deficits and late-emerging morphological deficits provides empirical support for both universal models of reading development (Share, 2025) and Hebrew-specific frameworks (Share & Bar-On, 2018), illustrating how the phonological-to-morphological shift manifests across dyslexia pathways. Third, we advance understanding of the long-term predictive power of kindergarten skills by demonstrating their distinct roles across Grades 1–4. Although often viewed as relevant mainly to initial acquisition, these skills exert sustained, stage-specific influence throughout reading development.

At the practical level, our findings underscore the necessity of comprehensive early screening that includes phonological awareness, letter knowledge, rapid naming, and morphological awareness to identify diverse dyslexia pathways, aligning with evidence supporting multi-indicator identification approaches (Wagner et al., 2020). Interventions should be tailored to children’s specific cognitive-linguistic profiles, recognizing that different pathways require distinct support strategies. Notably, morphological deficits may signal late-emerging difficulties that could otherwise be overlooked. We therefore emphasize incorporating morphological measures into early assessments and instructional programs across languages, even if related difficulties do not manifest until later grades. This preventive approach is particularly beneficial for children who exhibit early morphological weaknesses despite intact phonological skills, particularly in morphologically rich languages. Furthermore, our findings highlight the importance of ongoing monitoring through Grade 4 and differentiated, targeted support aligned with each child’s deficit profile. These insights should inform teacher training programs, equipping educators to recognize diverse dyslexia pathways and respond effectively, thereby optimizing educational outcomes.

While this study substantially advances understanding of dyslexia’s developmental pathways and early predictors, several limitations should be acknowledged. First, our sample comprised only Hebrew-speaking children, underscoring the need for cross-linguistic validation. In particular, replication studies should investigate whether morphological awareness serves as a universal predictor of late-emerging dyslexia across diverse linguistic contexts or whether its predictive utility is specific to morphologically rich languages like Hebrew. Second, although our composite morphological awareness measure (combining two derivational and one inflectional task) was supported by factor analysis as a unified construct (Cohen-Mimran et al., 2023) and proved highly predictive of late-emerging dyslexia (OR = 6.21), combining derivational and inflectional tasks may obscure theoretically and developmentally meaningful differences. Research in Hebrew indicates that derivational and inflectional morphology follow distinct acquisition timelines and cognitive demands (Ravid & Schiff, 2006), with derivational morphology being particularly critical for reading unpointed text and potentially more closely tied to mechanisms underlying late-emerging dyslexia. Future research should examine whether these morphological subtypes differentially contribute to pathway prediction, which could refine early identification strategies and clarify mechanisms underlying late-emerging reading difficulties. Third, the absence of a vocabulary measure limits interpretation, as vocabulary knowledge is deeply intertwined with morphological awareness, particularly derivational morphology. Poor performance on morphological tasks may partly reflect restricted lexical knowledge rather than purely morphological processing difficulties. Future research should include vocabulary assessment to disentangle these interrelated linguistic components and clarify whether morphological deficits represent independent risk factors or reflect broader language weaknesses.

## Conclusion

This study identified three distinct developmental pathways of dyslexia in Hebrew—persistent, resolving, and late-emerging—extending prior findings to a Semitic language. The higher resolving rates observed compared to Western languages suggest that morphological root-pattern transparency offers compensatory mechanisms for struggling readers beyond phonological grapheme-phoneme transparency, illustrating how typological features influence pathway prevalence. Furthermore, the study revealed distinctive patterns of kindergarten predictors for each pathway. Letter knowledge deficits emerged as a universal predictor across all pathways, while other skills showed pathway-specific associations. Phonological awareness deficits predicted both persistent and resolving dyslexia, while rapid naming deficits specifically predicted long-term persistent dyslexia, distinguishing it from the resolving pattern. Most notably, morphological awareness deficits uniquely marked late-emerging dyslexia with large effect sizes (OR = 6.21), a novel finding that addresses a critical theoretical and clinical gap in early identification efforts.

These findings support a multi-componential view of dyslexia and advance cross-linguistic frameworks for understanding its developmental nature by demonstrating how the universal phonological-to-morphological shift in reading manifests within dyslexia trajectories. Such insights refine theoretical models of reading and dyslexia and underscore the need for comprehensive kindergarten assessments that include morphological awareness alongside traditional screening measures, ultimately demonstrating how universal developmental mechanisms interact with language-specific features to guide effective educational practices across diverse linguistic contexts.

## Data Availability

Deidentified participant data will be available from the corresponding author upon reasonable request with a methodologically sound proposal. A data use agreement will be required.
